# Associations among loneliness, internal locus of control and subjective accelerated ageing in older adults who received the booster vaccination

**DOI:** 10.1192/bjo.2024.14

**Published:** 2024-02-26

**Authors:** Lee Greenblatt-Kimron, Yuval Palgi, Tali Regev, Boaz M. Ben-David

**Affiliations:** School of Social Work, Ariel University, Israel; Department of Gerontology, University of Haifa, Israel; School of Economics, Reichman University (IDC), Israel; Baruch Ivcher School of Psychology, Reichman University (IDC), Israel; Department of Speech-Language Pathology, University of Toronto, Canada; and KITE, Toronto Rehabilitation Institute, University Health Networks, Canada

**Keywords:** Loneliness, internal locus of control, subjective accelerated ageing, older adults

## Abstract

**Background:**

A rise in loneliness among older adults since the COVID-19 outbreak, even after vaccination, has been highlighted. Loneliness has deleterious consequences, with specific effects on perceptions of the ageing process during the COVID-19 pandemic. Coping with stressful life events and the challenges of ageing may result in a perception of acceleration of this process.

**Aim:**

Studies have shown a buffering effect of an internal locus of control in the relationship between COVID-19 stress and mental distress. The current study examined whether loneliness predicts subjective accelerated ageing and whether internal locus of control moderates this relationship.

**Method:**

Two waves of community-dwelling older adults (*M* = 70.44, s.d. = 5.95; age range 61–88 years), vaccinated three times, were sampled by a web-survey company. Participants completed the questionnaire after the beginning of the third vaccination campaign and reported again 4 months later on loneliness, internal locus of control and subjective accelerated ageing level in the second wave.

**Results:**

Participants with higher levels of loneliness presented 4 months later with higher subjective accelerated ageing. Participants with a low level of internal locus of control presented 4 months later with high subjective accelerated ageing, regardless of their loneliness level. Participants with a high level of internal locus of control and a low level of loneliness presented with the lowest subjective accelerated ageing 4 months later.

**Conclusions:**

The findings emphasise the deleterious effects of loneliness and low internal locus of control on older adults’ perception of their ageing process. Practitioners should focus their interventions not only on loneliness but also on improving the sense of internal locus of control to improve subjective accelerated ageing.

The consequences of the COVID-19 pandemic on older adults have been widely explored in research, with older adults known to be most at risk worldwide^1^ and to be psychologically affected even after receiving vaccination.^2^ Preliminary studies have demonstrated adverse psychological outcomes among older adults linked with the pandemic,^3^ including depressive symptoms, anxiety, worries, stress,^4^ death anxiety,^5^ peritraumatic distress^6^ and ageism.^7^ Loneliness was recognised as one of the most deleterious factors that affected older adults’ mental health during the pandemic.^4,8^ Specifically, it was demonstrated that loneliness was related to a higher level of depressive symptoms, anxiety and their comorbidity during the COVID-19 outbreak.^9^ Vaccination programmes were supposed to be the ‘light at the end of the pandemic tunnel’ with respect to the adverse consequences of COVID-19.^10^ Despite this, associations have been found between physical side-effects of vaccination and depression in older adults.^10^ In addition, vaccination hesitancy was associated with anxiety, depression and peritraumatic stress disorder among older adults who had received vaccinations.^2^ Greenblatt-Kimron and colleagues^11^ found that older adults were sensitive to distinctive factors linked with clinical depression, which were affected by their world assumptions during COVID-19 vaccination programmes. The authors concluded that the elevated level of depression following vaccination suggests that it may take time to recuperate from depression linked to pandemic distress. Notably, even the termination of nationwide social restrictions was not sufficient to alleviate their detrimental psychological effects.^12^ Based on the studies mentioned above that examined various mental health variables,^2,10,11^ we assumed that a similar pattern would occur concerning ageing perceptions with the variables in the current study, which had yet to be examined.

Coping with stressful life events, together with the challenges of ageing, may result in a perception of acceleration in the ageing process, as related to adverse psychological outcomes.^6,13^ Therefore, the current study aimed to examine the longitudinal relationship between loneliness and subjective accelerated ageing of older adults during the booster vaccination programme in Israel and social restriction policy. Internal locus of control is one of the factors found to buffer the relationship between COVID-19 stress and general mental distress.^14^ In addition, a link was found between health locus of control and views of ageing.^15^ Accordingly, the current study aimed to examine whether internal locus of control moderates the link between loneliness and subjective accelerated ageing among older adults during the booster vaccination campaign.

## Loneliness and subjective accelerated ageing

Loneliness, described as the perception of unsatisfied personal, emotional and social needs for company,^16^ has been related to accelerated biological and/or physical ageing.^17^ During the COVID-19 pandemic, loneliness was shown to increase among older people.^18^ In turn, loneliness during the COVID-19 pandemic was associated with negative psychiatric symptoms among older adults; however, this relationship was buffered by favourable perceptions of ageing.^4^

It was recently suggested that in the second half of life, individuals construct their personal perceptions regarding the ageing process and tend to evaluate how fast or slow they are ageing. In other words, they become aware of the rate at which they are ageing, an evaluation known as subjective accelerated ageing.^13^ Previous studies showed a link between faster subjective accelerated ageing and negative social aspects of ageing such as ageism,^19^ as well as negative aspects of mental health such as peritraumatic distress, worries and post-traumatic stress disorder (PTSD) symptoms.^6,13^

Subjective accelerated ageing was included as one of the measures in the concept of personal views of ageing.^20^ As such, this concept refers to individuals’ representations regarding their own ageing process and the state of being old. More specifically, it assumes that individuals tend to cognitively evaluate an internalised clock and construct an identity based on whether the rate at which their ageing is advancing is fast, normal or slow. The foundation of this theoretical concept is the idea that alongside the biological ageing process, there is a similar psychological process that is affected by social and internal factors such as loneliness and locus of control.

## Internal locus of control

Locus of control describes the premise that events are controlled internally or externally.^21^ People with an internal locus of control maintain that they are responsible for shaping and managing their lives, whereas those with an external locus of control suppose that their lives are controlled by luck, fate, opportunities, other people and events.^22^ Studies have shown links between high internal locus of control and favourable outcomes, such as lower levels of pain and better physical functioning.^23^ In particular, among older adults, studies have shown positive relationships of internal locus of control with higher self-reported health^24^ and better quality of life,^25^ whereas a negative relationship was found between internal locus of control and suicide.^26^ During the pandemic, high internal locus of control was found to buffer the link between COVID-19 stress and general mental distress.^14^ Moreover, higher internal locus of control was associated with lower depression and less stress.^27^ Higher internal locus of control was also linked with higher positive affect and with lower negative affect and psychological distress.^28^

## The current study

The study was conducted in two waves, following the first and second COVID-19 booster vaccination campaigns in Israel, 4 months apart. The study focused on older adults who received both booster vaccinations, which were assumed to also serve as psychological boosters. Based on the literature presented above, the present study aimed to assess whether loneliness in the first wave (W1; when severe social restrictions were imposed) was linked with subjective accelerated ageing in the second wave (W2), and whether internal locus of control, assessed in W1, moderated this link.

It was first hypothesised that higher levels of loneliness in W1 would be related to faster subjective accelerate ageing among older adults 4 months later. In addition, a higher level of internal locus of control in W1 would be related to slower subjective accelerate ageing in W2. The second hypothesis maintained that internal locus of control would moderate this association. Namely, older adults with higher levels of internal locus of control in W1 would show a stronger positive relationship between loneliness and subjective accelerated ageing in W2 (i.e. reduced loneliness leading to reduced subjective accelerated ageing), compared with older adults with lower levels of internal locus of control.

## Method

### Participants and sampling design

We conducted an *a priori* power analysis using G*Power (version 3.1)^29^ to detect whether there was a significant association using a linear bivariate regression as a close approximation for the model used. We chose a H1 slope coefficient (0.2). Therefore, a sample size of at least 262 participants was the minimal requirement for an actual power of 0.95. To account for attrition in a longitudinal study (about a third), additional participants were recruited, resulting in 400 participants, a number that also fit within our budgetary constraints. W1 was collected from an Israeli online panel of responders during 11–12 August 2021, about 2 weeks after the beginning of the booster vaccination campaign. Participants were all Jewish and Israeli older adults (*M* = 69.44, s.d. = 5.95, range = 60–87), about half of whom were men (54.1%), and eligible for receiving the booster vaccine. Data were collected by a web-based online survey company (https://www.midgam.co.il/About-en/). This online panel has access to tens of thousands of Israelis who are interested in participating in online studies, in exchange for monetary reimbursement. The survey company published the survey for all eligible panel members (~2% of the online panel, more than 1000 members) and closed the study 1 day after publishing it, once the quota of 400 participants had completed the questionnaire: 200 aged 60–69 years and 200 aged 70–80. All participants were reimbursed for their participation, as reported by the survey company, and all 400 participants who responded to the survey were included.

W2 was conducted about 5 months later, on 5–6 January 2022, about 2 weeks after the beginning of the vaccination campaign for the fourth (booster) vaccination. In August 2021, Israel was the first country to administer the first vaccine for COVID-19 for individuals aged 60 years and over; accordingly, on 2 January 2022, Israel was the first country to approve the second vaccine. Only 295 (73.8%) participants from W1 agreed to participate in W2. Twenty-seven participants from W1 were removed as they did not receive the third booster vaccination and therefore were not eligible for the fourth booster vaccination. Data were collected by the same online survey company (MIDGAM). It is notable that at that time, the majority of older adults in Israel had received the COVID-19 vaccination (https://datadashboard.health.gov.il/COVID-19/general). Thus, our sample was selected to resemble vaccination status in Israel and avoid confounding variables, such as age and disease, which may have resulted in participants reporting subjective accelerated ageing due to factors related to health and cognition rather than the vaccination and which may have led to vaccine refusal.

Attrition analysis showed that participants who only completed W1 were not different from those who completed both waves in terms of demographics: age, *t*(398) = 1.369, *P* = 0.172; gender, *ꭓ^2^*(1) = 2.208, *P* = 0.137; college education, *ꭓ^2^*(1) = 0.666, *P* = 0.416; and marital status, *ꭓ^2^*(1) = 0.180, *P* = 0.671.

The authors assert that all procedures contributing to this work comply with the ethical standards of the relevant national and institutional committees on human experimentation and with the Helsinki Declaration of 1975, as revised in 2008. All procedures involving human subjects/patients were approved by the institutional review board of the Reichman University (IDC), Herzliya, Israel (reference number P_2021138). Data were anonymised by the survey company before being sent to the research team. No name or personal details were given, and all personal information was secured by the survey company following its rules and regulation. Informed consent (as approved by P_2021138) was obtained electronically from all participants, as a mandatory condition before survey commencement. Specifically, informed consent was provided by the participant choosing an option on the electronic form stating that they had read the instructions and confirmed consent to participate in the study.

### Measures

Demographics were all measured in W1. Demographic factors included: age, gender, marital status (1 = single, divorced or widowed; 2 = married or living with a partner) and years of education (1 = non-academic, 2 = academic). For the complete demographic data of participants, please refer to Table 1. Covariates included: self-rated health, which was measured with a single item ‘how would you rate your health in the last month?’, on scales ranging from 1 (not good at all) to 5 (very good).^30^ Being in a risk group was measured by a 1 = yes, 2 = no single item asking if the participant had been diagnosed with a chronic condition related to increased risk of death due to COVID-19 complications (e.g. cardiovascular disease, diabetes). In W2, participants were asked whether they had received the fourth booster vaccine (1 = no, 2 = yes).

**Table 1:** Participants’ demographic characteristics (*N* = 268)

Demographic characteristics	*N* (%)
Gender (%)	
Male	123 (45.9)
Female	145 (54.1)
Mean age in years (s.d.), range	70.44 (5.95), 61–88
Marital status (%)	
Married	194 (72.4)
Not married	74 (27.6)
Academic education	141 (52.6)
Risk group owing to chronic diseases related to COVID-19 risk	153 (57.1)
Received fourth booster vaccination in first week of campaign	66 (24.6)
Mean subjective rated health (s.d.), range	3.77 (1.00), 1–5

Internal locus of control was measured using three items from the validated KMKB scale (German: Kurzskalen zur Messung von Kontrollüberzeugungen in Bevölkerungsumfragen)^31^ in W1 to form a three-item short subscale.^32^ Participants were asked to what degree they agree with the following statements: ‘I like taking responsibility’, ‘I find it best to make decisions myself, rather than to rely on fate’ and ‘When I encounter problems or opposition, I usually find ways and means to overcome them’. Items were rated on a scale ranging from 1 (applies to me to a very great extent) to 5 (does not apply to me at all). Ratings on all items were averaged to create a score (as suggested by the KMKB scale^32^) with a range of 1 (very low) to 5 (very high). The internal reliability for this scale was α = 0.844.

Loneliness was measured with a validated three-item short version of the UCLA (University California Los Angeles) loneliness scale (α = 0.931),^33^ with items rated on a five-point scale ranging from 1 (not at all) to 5 (almost always), in W1. The score was the sum of ratings across items (as suggested by the UCLA scale^33^), with a range of 3 (very low) to 15 (very high) loneliness.

Subjective accelerated ageing^13^ was measured with a single item, ‘In general, I feel that my ageing rate is’, on a scale ranging from 1 (very slow) to 5 (very fast), in W2. Higher scores indicated higher subjective accelerated ageing. Subjective accelerated ageing in W1 was controlled. The use of this single item has been found to be reliable in previous studies.^6,13,19^

### Data analysis

First, we conducted Pearson correlation analysis for the relevant variables to examine general associations between study variables. Second, we conducted multiple hierarchical regression to examine the specific multi-relations among the study variables. Third, significant interactions were investigated with the PROCESS computational tool^34^ to test the significance of simple slopes at two different levels (−1 and +1 s.d.) from the mean internal locus of control level. Demographic characteristics and covariates (age, gender, years of education, marital status, self-rated health, being in a risk group and subjective accelerated ageing in W1; and whether the participant had received the fourth vaccination as reported in W2) were entered in the first step of the multiple hierarchical regression. These additional demographic measures were used to control for potential biases in the sample and potential effects on the dependent measure.^35–37^ Levels of loneliness and internal locus of control as reported in W1 were entered in the second step. In the third step, the interaction between loneliness and internal locus of control predicted subjective accelerated ageing in W2.

## Results

Overall, 268 of the original 400 participants from W1 completed W2. Most of the participants in the final sample were married or cohabiting (72.4%), about half (52.6%) had had an academic education, and 57.1% reported belonging to a risk group owing to chronic diseases related to risk for COVID-19. In this sample, 24.6% reported that they received the fourth booster vaccination in the first week of the campaign.

Table 2 presents the data and intercorrelations of the study variables. Loneliness was positively correlated with subjective accelerated ageing (*r* = 0.32, *P* < 0.001), and internal locus of control was negatively correlated with subjective accelerated ageing (*r* = −0.15, *P* < 0.05). Loneliness was not significantly correlated with internal locus of control (*r* = −0.10, *P* = 0.11).

**Table 2:** Means, standard deviations, ranges and Pearson correlations for the study variables (*N* = 268)

Variables	1	2	3	4
1. Locus of control wave 1	–			
2. Loneliness wave 1	−0.10	–		
3. Subjective accelerated ageing wave 1^a^	−0.12*	0.36***	–	
4. Subjective accelerated ageing wave 2^a^	−0.15*	0.32***	0.56***	
Mean	4.21	6.55	2.67	2.63
s.d.	0.61	3.17	0.96	0.91
Range	1–5	3–15	1–5	1–5

a. Higher scores indicate that the participant felt a higher level of accelerated ageing.

*P < 0.05, ***P < 0.001.

Next, following our first hypothesis, we conducted a hierarchical regression analysis to examine the moderating role of internal locus of control in W1 on the relationship between loneliness in W1 and subjective accelerated ageing in W2. Subjective accelerated ageing in W1 was controlled for. In the first step, the demographic factors (age, gender, marital status and education) and covariates (self-rated health, being in a risk group, all in W1, and whether the participant had received the fourth vaccination) were entered. Loneliness and internal locus of control in W1 were entered in the second step. The results showed that those who reported a higher level of loneliness (*B* = 0.03, β = 0.12, *t*[256] = 2.05, *P* < 0.05) also reported higher feelings of subjective accelerated ageing in W2. Internal locus of control was not significantly related to subjective accelerated ageing in W2 after controlling for demographics (*B* = −0.08, β = −0.06, *t*[256] = −0.97, *P* = 0.34).

Following the second hypothesis, the interaction between loneliness and internal locus of control in W1 was entered in the third step, and the results showed that participants with a higher level of internal locus of control demonstrated a stronger association between loneliness and subjective accelerated ageing (*B* = 0.07, *t*[257] = 2.99, *P* < 0.005). This interaction accounted for an additional 2% of the variance in subjective accelerated ageing.

**Fig. 1:**
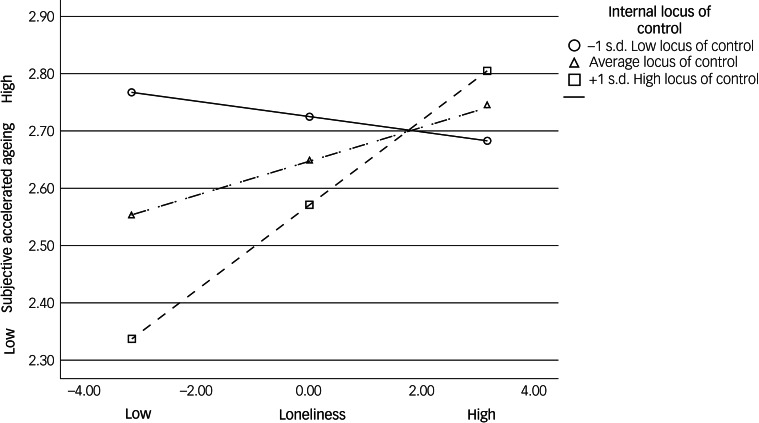
Moderating effect of internal locus of control on the association between loneliness (centred) and subjective accelerated ageing.

To estimate the effects of simple slopes at two different levels (high and low; ±1 s.d. from the mean) of internal locus of control, we used Hayes’ computational procedure.^34^ This analysis showed that for individuals who reported an internal locus of control in W1 that was 1 s.d. below the mean (low internal locus of control), each additional loneliness score in W1 was associated with a non-significant decrease of −0.01 points in subjective accelerated ageing in W2 (*B* = 0.01, *t*[256] = −0.56, *P* = 0.57). For individuals who reported an internal locus of control level that was 1 s.d. above the mean (high internal locus of control), each additional loneliness score was associated with a significant increase of 0.07 points in the level of subjective accelerated ageing (*B* = −0.07, *t*[256] = 3.51, *P* < 0.001). These trends are visualised in Fig. 1.

## Discussion

To the best of our knowledge, the present study is the first to investigate the role of internal locus of control in moderating the association between loneliness and subjective accelerated ageing among older adults during the COVID-19 booster vaccination programmes. It is also one of the few studies to generally test these links longitudinally. As hypothesised, older adults who reported higher levels of loneliness reported more subjective accelerated ageing 4 months later. Also following our hypothesis, internal locus of control was found to moderate this link. Namely, a lower level of loneliness was associated with slower perceived accelerated ageing 4 months later only for participants with high levels of internal locus of control. Participants with lower levels of internal locus of control perceived their ageing process as advancing faster (when tested 4 months later), regardless of their levels of loneliness.

In line with the first hypothesis, a positive relationship was found between loneliness and subjective accelerated ageing, measured 4 months later. This relationship supports the findings of previous studies highlighting the physical and psychological distress of older adults during a pandemic.^6^ Together, these findings emphasise the impact of stressful life events on the subjective ageing process. The results could also be related to the link reported between subjective age and neurobiological accelerated ageing,^38^ as well as between PTSD symptoms and subjective accelerated ageing.^13^ Nevertheless, loneliness was not significantly associated with internal locus of control. This may be explained by internal locus of control being a trait-like characteristic and, therefore, possibly less affected by fluctuations associated with the experience of loneliness, as occurred during the coronavirus pandemic, especially for the older population. With regard to the booster vaccination, previous results in Israel showed that attitudes regarding the COVID-19 vaccination were more negative compared with attitudes regarding other vaccines.^39^ On this basis, having an internal locus of control may be more dominant than the ‘light at the end of the pandemic tunnel’,^8^ namely the promised results of the booster vaccination.

Supporting the second hypothesis, participants in the current study with low levels of internal locus of control reported high levels of subjective accelerated ageing, regardless of their level of loneliness. By contrast, participants with a high level of internal locus of control reported the slowest subjective accelerated ageing with low levels of loneliness and fast subjective accelerated ageing with high level of loneliness. This may be explained by people with a high level of internal locus of control believing that they are responsible for determining their lives^22^ and supports previous findings during the COVID-19 pandemic showing that a high internal locus of control buffered the relationship between COVID-19 stress and general mental distress.^14^

The present study had limitations. First, data were collected through a web-based online survey company and may have been biased towards older adults exposed to this network. We acknowledge that these participants are likely to be more technologically savvy than the average older adult population. Second, the study was based on self-report questionnaires, which may limit the ability to generalise the findings. Third, the measures were assessed by very short scales. Fourth, one may consider using subjective accelerated ageing as a moderator and not as the dependent variable. However, our analysis closely follows models from previous pertinent studies.^13,19^ Future studies may wish to further explore other models. Fifth, we focused on older adults who received the COVID-19 vaccine. Even though vaccine adoption was very high for older adults in Israel (>90%), it is possible that non-adopters may have been differently affected by the study measures owing to medical reasons and, therefore, may have reported faster subjective accelerated ageing owing to health problems. In addition, although the rationale for studying the vaccinated sample in the current study was that vaccination side-effects have associations with psychological risk factors, the current study did not include variables on vaccine side-effects or data on depression, anxiety and peritraumatic stress. We recommend that future longitudinal studies incorporate these data in their examination of the relationships among locus of control, loneliness and subjective accelerated ageing. Finally, there are no data regarding response rates, also limiting the generalisability of the findings.

Despite these limitations, the present study is the first to examine the relationship between loneliness and subjective accelerated ageing during the COVID-19 pandemic among older adults and the moderating role of internal locus of control in this relationship. The findings have theoretical and practical implications.

Theoretically, the study emphasises that higher levels of internal locus of control allow older adults to reap the gains of a lower sense of loneliness. This suggests that when personal resources are internal, individuals may feel more in control after being vaccinated. When loneliness levels are low, this could ultimately have a positive effect on the personal perception of the self-ageing process. These results are also in line with recent data from our laboratory indicating that adoption of COVID-19 booster vaccinations can be related to an increased sense of control owing to the vaccine and reduced anxiety.^40,41^

On a practical level, the rise in loneliness among older adults since the COVID-19 outbreak cannot be understated.^4^ Our results suggest that loneliness can have severe deleterious effects even 4 months later. However, it appears that reducing loneliness is a precondition for the gains of higher locus of control to be reaped. In this regard, the results provide evidence of the need for suitable interventions aimed at reducing loneliness before other harmful aspects of ageing can be tackled. For example, a recent study suggested that inducing interpersonal synchrony, which was lacking during social restrictions, could increase positive mood and perceived partner responsiveness in older adults.^42^ Similarly, closeness or even just presenting the picture of a loved one (a security-enhancing figure) was found to improve auditory perception in older age,^43^ related to effective spoken communication, cognitive processing and active ageing.^36,44,45^ Digital literacy interventions were also found to be effective,^46,47^ specifically when interventions are designed to meet older adults' goals and sensory abilities.^48,49^

In summary, this study emphasises the adverse outcome of loneliness on older adults’ perception of their ageing process. These effects are accentuated during stressful life events, such as the COVID-19 pandemic, even after the implantation of vaccination programmes. Findings show that low levels of loneliness together with a high internal locus of control provide a recipe for a slower pace of perceived accelerated ageing, which can be detected 4 months later. Interventions should be tailored first to reduce loneliness as a precondition and then to enhance the perception of an internal locus of control.

The current findings suggest that loneliness should not be tackled on its own but in combination with internal locus of control in older age. First, this points to the important of assessing locus of control. Second, promoting higher internal locus of control should be seen as a goal for intervention in older age. This is of special importance in times of stressful life events.^6^

## Data Availability

Raw data are available upon request from the corresponding author (B.M.B.-D.).
